# 5-hydroxymethylcytosine sequencing in plasma cell-free DNA identifies unique epigenomic features in prostate cancer patients resistant to androgen deprivation therapy

**DOI:** 10.1101/2023.10.13.23296758

**Published:** 2023-10-16

**Authors:** Qianxia Li, Chiang-Ching Huang, Shane Huang, Yijun Tian, Jinyong Huang, Amirreza Bitaraf, Xiaowei Dong, Marja T. Nevalanen, Jingsong Zhang, Brandon J. Manley, Jong Y. Park, Manish Kohli, Elizabeth M. Gore, Deepak Kilari, Liang Wang

**Affiliations:** 1 Department of Tumor Biology, H. Lee Moffitt Cancer Center and Research Institute, Tampa, Florida, USA; 2 Department of Oncology, Tongji Hospital, Huazhong University of Science and Technology, Wuhan, China; 3 Department of Biostatics, Joseph J. Zilber College of Public Health, University of Wisconsin, Milwaukee, Wisconsin, USA; 4 Department of Biostatics, University of Wisconsin, Madison, Wisconsin, USA; 5 Dept. of Pharmacology, Physiology and Cancer Biology, Sidney Kimmel Cancer Center, Thomas Jefferson University, Philadelphia, USA; 6 Department of Genitourinary Oncology, H. Lee Moffitt Cancer Center and Research Institute, Tampa, Florida, USA; 7 Department of Cancer Epidemiology, H. Lee Moffitt Cancer Center and Research Institute, Tampa, Florida, USA; 8 Department of Oncology, Huntsman Cancer Center, University of Utah, Salt Lake City, Utah, USA; 9 Department of Radiation Oncology, Medical College of Wisconsin, Milwaukee, Wisconsin, USA; 10 Division of Oncology, Medical College of Wisconsin, Milwaukee, Wisconsin, USA

**Keywords:** metastatic hormone sensitive prostate cancer, androgen deprivation therapy, 5-hydroxymethylcytosine, cell-free DNA, androgen receptor, liquid biopsy

## Abstract

**Background:**

Currently there are no biomarkers to identify resistance to androgen-deprivation therapy (ADT) in men with hormone-naive prostate cancer. 5-hydroxymethylcytosines (5hmC) in the gene body are associated with gene activation and are critical for epigenomic regulation of cancer progression.

**Objective:**

To evaluate whether 5hmC signature in cell-free DNA (cfDNA) predicts early ADT resistance.

**Design, Setting, and Participants:**

Serial plasma samples from 55 prostate cancer patients receiving ADT were collected at three timepoints including baseline (prior to initiating ADT, N=55), 3-month (after initiating ADT, N=55), and disease progression (N=15) within 24 months or 24-month if no progression was detected (N=14). 20 of the 55 patients showed disease progression during the 24-month follow-up. The remaining 35 patients showed no progression in the same follow-up period.

**Outcome Measurements and Statistical Analysis:**

cfDNA (5–10ng) was used for selective chemical labeling (hMe-Seal) sequencing to map 5hmC abundance across the genome. Read counts in gene bodies were normalized with DESeq2. Differential methylation and gene set enrichment analyses were performed to identify the 5hmC-enriched genes and biological processes that were associated with disease progression. Kaplan-Meir analysis was utilized to determine the association of 5hmC signatures with progression-free survival.

**Results and Limitations:**

5hmC-sequencing generated an average of 18.6 (range 6.03 to 42.43) million reads per sample with 98% (95–99%) mappable rate. Baseline sample comparisons identified significant 5hmC difference in 1,642 of 23,433 genes between 20 patients with progression and 35 patients without progression (false discovery rate, FDR<0.1). Patients with progression showed significant enrichments in multiple hallmark gene sets with androgen responses as the top enriched gene set (FDR=1.19E-13). Interestingly, this enrichment was driven by a subgroup of patients with disease progression featuring a significant 5hmC hypermethylation of the gene sets involving *AR*, *FOXA1* and *GRHL2*. To quantify overall activities of these gene sets, we developed a gene set activity score algorithm using a mean value of log2 ratios of gene read counts in an entire gene set. We found that the activity scores in these gene sets were significantly higher in this subgroup of patients with progression than in the remaining patients regardless of the progression status. Furthermore, the high activity scores in these gene sets were associated with poor progression-free survival (p <0.05). Longitudinal analysis showed that activity scores in this subgroup with progression were significantly reduced after 3-month ADT but returned to high levels when the disease was progressed.

**Conclusions:**

5hmC-sequencing in cfDNA identified a subgroup of prostate cancer patients with preexisting activation (5hmC hypermethylation) of gene sets involving *AR*, *FOXA1* and *GRHL2* before initiating ADT. Activity scores in these gene sets may serve as sensitive biomarkers to determine treatment resistance, monitor disease progression and potentially identify patients who would benefit from upfront treatment intensification. More studies are needed to validate this initial finding.

**Patient summary:**

There are no clinical tests to identify prostate cancer patients who will develop early resistance to androgen deprivation therapy within 24 months. In this study, we evaluated cell-free DNA epigenomic modification in blood and identified significant enrichment of 5-hydroxymethylation in androgen response genes in a subgroup of patients with treatment resistance. High level 5-hydroxylmethylation in these genes may serve as a discriminative biomarker to diagnose patients who are likely to experience early failure during androgen deprivation therapy.

## Introduction

Prostate cancer is the most common cancer in men and the second leading cause of cancer-related death[1]. Prostate cancer cells are initially reliant on circulating androgens to activate endogenous androgen receptor (*AR*)[2]. Suppression of testicular androgens by castration (medical or surgical) is the mainstay of treatment for metastatic disease[3]. Androgen deprivation therapy (ADT) achieves a remission in 80–90% of men with advanced prostate cancer and an average progression-free interval of 12–33 months[4]. Despite the efficacy of ADT, significant challenges persist. ADT is not curative and disease progresses in virtually all patients[5]. To address this issue, the therapeutic strategy has evolved with the addition of other systemic agents. The combination of ADT with Docetaxel or with new androgen receptor signaling inhibitors (ARSI) has shown substantial benefits [6]. Because docetaxel and ARSI have different mechanisms of action on androgen signaling and prostate cancer cells, the combination may enhance the treatment effect[7–9]. However, patients with similar clinical pathological factors may respond differently, suggesting phenotypic heterogeneity and potential role of genetic background in the treatment response. To offer more effective treatment, it will be essential to understand which patient group would truly benefit from this therapy. Development of biomarkers will facilitate the selection of the most appropriate treatment.

Currently, tissue biopsies have demonstrated a limited role outside of histologic diagnosis for treatment decision and are impractical to perform routinely in clinical practice due to the bone-predominant metastasis of prostate cancer. To address this challenge, recent research focus has turned to the development of minimally-invasive biomarkers from body fluids [10]. Blood serological biomarker prostate specific antigens (PSA) is commonly used for screening and diagnosis of prostate cancer, as well as for monitoring treatment response [11]. However, PSA does not predict systemic treatment response. In search for more predictive biomarkers, cell-free DNA (cfDNA) circulating in blood has attracted significant attention[12]. Minimally invasive detection of somatic variations using blood samples offers substantial advantages over tissue biopsy as it can detect entire genetic makeup from tumor tissues. The easily accessible nature of blood makes it an ideal sample source for real-time and dynamic monitor of the treatment response and disease progression[13–15].

DNA methylation at certain genomic regions is characteristic of human cancers and has been used as a specific biomarker for cancer detection and outcome prediction [16]. In addition to commonly reported 5-methylcytosines in the genome, 5-hydroxymethylcytosines (5hmC) are also abundant. The 5hmC is generated through oxidation of 5-methylcytosines by the ten-eleven translocation enzymes [17]. Recent genome-wide sequencing maps of 5hmC in various mammalian cells and tissues support its regulatory role for gene expression [18–25]. The 5hmC is enriched at transcriptionally active regions, such as gene-bodies, promoters and enhancers [24, 26]. It may represent dynamically activated transcription rather than constitutively expressed house-keeping genes [27]. Therefore, 5hmC has emerged as a novel class of cancer epigenomic biomarkers [16, 25, 28–33], with a recent study showing cfDNA 5hmC as a prognostic factor in metastatic prostate cancer [25]. However, this study used blood sample from one time point only and examined 5hmC for its potential role in prognosis. So far, little is known about the potency and reliability of the cell-free 5hmC as a predictive biomarker of treatment response for metastatic hormone-sensitive prostate cancer under ADT and its combination therapy. To address this question, we employed the 5hmC-Seal [20], a highly sensitive and selective chemical labelling-based sequencing technology, to profile the full spectrum of 5hmC in the hormone-sensitive patients with three time points of blood collection. Our results reveal that 5hmC-based epigenomic features are associated with treatment response and classify early resistance to ADT.

## Patients and Methods

### Patient cohort

A total of 55 prostate cancer patients were enrolled in the study. Among those, 30 patients were recruited from the Medical College of Wisconsin and additional 25 patients were recruited from the Milwaukee VA Medical Center, United States. All patients were diagnosed with advanced prostate cancer and met the NCCN treatment guideline for ADT. Blood was collected before initiating ADT (baseline), 3 months after initiating ADT and 24 months after ADT or at disease progression within the 2-year follow-up. Written informed consent was obtained from all participants prior to enrolment. Progression was defined as either radiological (as defined by Prostate Cancer Working Group 2 criteria)[34] or clinical (defined as worsening disease-related symptoms necessitating a change in anti-cancer therapy and/or deterioration in ECOG performance status ≥2 levels)[35]. Additionally, plasma samples and peripheral blood mononuclear cells (PBMCs) from 8 healthy subjects were also collected.

### Blood sample processing

Plasma was prepared within 2 hours after blood draw. Collected blood samples were first centrifuged at 1000g for 10 minutes at RT to separate plasma. The platelets-rich plasma was immediately centrifuged again at 5000g for additional 10 minutes to collect platelets-poor plasma before stored at −80°C. cfDNAs were extracted from 400–1000μl of platelets-poor plasma using QIAamp DNA Blood Mini Kit (Qiagen). Final DNA eluent (50μl) was quantified by a Qubit 2.0 Fluorometer (Life Technology) and stored at −80°C until use.

### Spike-in controls for 5hmC enrichment

The 5hMe-Seal method was previously published[20, 29]. In brief, the spiked-in control was generated by PCR-amplified lambda DNA using a cocktail of dATP/dGTP/dTTP and one of the following: dCTP, dmCTP or 10% dhmCTP (Zymo)/90% dCTP. Primers sequences are as follows: dCTP FW-5′-CGTTTCCGTTCTTCTTCGTC-3′, RV-5′ TACTCGCACCGAAAATGTCA-3′; dmCTP FW-5′-GTGGCGGGTTATGATGAACT-3′, RV-5′-CATAAAATGCGGGGATTCAC-3′; 10% dhmCTP/90% dCTP FW-5′-TGAAAACGAAAGGGGATACG-3′, RV-5′-GTCCAGCTGGGAGTCGATAC-3′. The spike-in probes were utilized to monitor the robustness and sensitivity of 5hmC-Seal. In total, 0.2 million copies of 5hmC and no 5hmC spike-ins were first mixed and then added into cfDNA samples before library preparation. The input control samples omitted the 5hmC pull-down step to generate non-5hmC enrichment libraries. After sequencing, spike-in reads were called and enrichment ratios were calculated.

### 5hmC library construction and high-throughput sequencing

5hmC libraries were constructed as described previously[29]. Briefly, the cfDNA was ligated with sequencing adaptors. The ligated DNA was incubated in a 25ul reaction solution containing HEPES buffer (50 mM, pH 8.0), MgCl2 (25 mM), N3-UDP-Glc (100 mM, Active Motif), and 12.5 U T4 phage β-glucosyltransferase (βGT, Thermo Fisher) for 1 h at 37 °C. Then, 2.5μl DBCO-PEG4-biotin (20 mM stock in DMSO, Click Chemistry Tools) was directly added to the reaction mixture and incubated for 2 h at 37°C. Subsequently, the DNA Clean & Concentrator^™^−5 (ZYMO Research) was used to purify the DNA. Thereafter, the purified DNA was incubated with C1 streptavidin beads (5ul, Life Technologies) for 15 min at room temperature. The beads subsequently underwent eight 5-min washes with buffer (5mM Tris pH 7.5, 0.5 mM EDTA, 1 M NaCl and 0.1% Tween 20). All binding and washing were done at room temperature with gentle rotation. Beads were then resuspended in water and amplified with 12–15 (cfDNA) or 9 (whole blood genomic DNA) cycles of PCR amplification using KAPA Library Amplification Kit (Kapa Biosystems). A separate non-enrichment control library from cfDNA was also made by direct PCR amplification from ligated DNA without labeling and capture. The amplified products were purified using AMPure XP beads and used for high-throughput 75 cycle single end sequencing on the Illumina NextSeq 500 platform.

### Data processing and normalization

The raw 5hmC-Seal data were first cleaned for adaptor sequences, followed by aligning to the human genome (hg19)[30]. Enrichment regions were identified by model-based analysis of ChIP-Seq (MACS) [36]. The read counts for either 1,000bp bins or individual genes were extracted using bedtools [37] and further normalized using DESeq2 [38], which performs the variance-stabilizing transformation to correct for sequencing depth and library size.

### Calculation of gene set activity scores

Gene lists of selected gene sets were downloaded from three data sources. The hallmark androgen response gene set was derived from GSEA website (https://www.gsea-msigdb.org/gsea/index.jsp). Lists of co-expressed genes with *AR*, *FOXA1*, and *GRHL2* as well as *AR* targets by ChIP-seq were downloaded from Enrichr gene set library [39]. The list of androgen signaling genes was copied from the publication [40]. To calculate gene set activity score, we transformed read counts into log⁡2 ratio of read counts in each individual genes between each patient and a pooled healthy control. We then identified mean value of the log⁡2 ratios in the gene set and further multiplied the mean value by 100. The formula is the following:

$$Activity\;Score\;=100\;\text{*}\;\left(\sum_{i=1}^{n}log_{2}\left(RC_{i}^{P}\right)-log_{2}\left(RC_{i}^{C}\right)\right)/n$$

where $RC_{i}^{P}$ is the normalized read count of gene i for a patient, $RC_{i}^{C}$ is the average normalized read count of gene i among the 7 healthy controls, and n is the total number of genes in a gene set. Normally, the activity score is near zero if the tested gene set in patients has similar 5hmC methylation level to healthy controls tested in the study. A positive score indicates activation (5hmC hypermethylation) of the gene set or signaling pathway.

### Statistical analyses

DESeq2 and t-tests were utilized to identify the epigenomic features associated with clinical outcomes. Kaplan Meir analysis was used to identify epigenomic features that were associated with progression-free survival. Analysis of covariance was applied to adjust for the effect of clinical covariates. For global analysis, false discovery rate (FDR) was used to correct for multiple testing with FDR less than 0.1 being used as cutoff. For differences in gene set activity scores, a p value less than 0.05 was considered as significant.

## Results

### Clinical characteristics and plasma collection

In the 55 advanced prostate cancer patients, the median age at enrollment was 69 (range 49–94). These patients included 44 Caucasian, 9 African Americans, 1 Asian, and 1 Native American. The number of patients with Gleason score of 6, 7, ≥ 8 and unknown were 2, 21, 30 and 2, respectively. The median baseline PSA was 21.5ng/ml (0.29–3275ng/ml). At enrollment, 44 of the 55 patients showed bone or soft tissue metastasis and the remaining 11 presented with non-metastatic castrate status (biochemical recurrence). Among the 44 patients with metastasis, 18 had high volume and 26 had low volume disease (CHAARTED criteria). Additionally, 28 of 55 patients were treatment-naïve at enrollment and the remaining 27 patients had prior treatment including 7 who underwent radical prostatectomy (RP), 8 radiotherapy (RT) and 12 both RP and RT. During 24-month follow-up, 20 patients showed disease progression (defined as early resistance to ADT in this study) and 35 had no sign of disease progression. Among the 20 patients with progression, the median time from ADT start to failure (clinical/radiographic progression) was 12.53 months (5.6–23.2 months). Metastatic sites and treatment information along with clinical characteristics are summarized in Table 1 (Supplementary Table S1 for detail).

**Figure 1.**
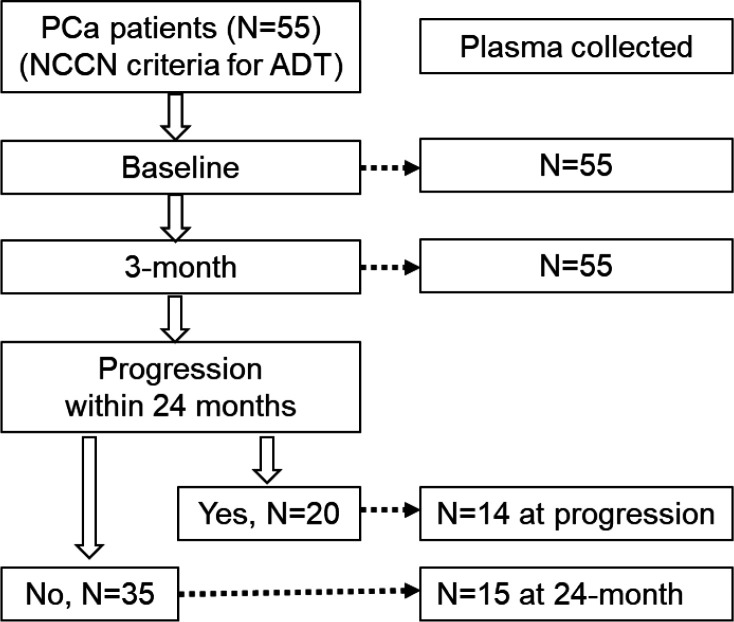
Study design and blood collection. A total of 55 patients who met the criteria for ADT were enrolled. Blood was collected at baseline (N=55), 3-month (N=55), at disease progression (N=14) and 24-month if no progression (N=15).

Baseline plasma samples (prior to ADT) and 3-month post ADT were collected in all 55 patients. Additional plasma samples were collected either at the time of progression in 14 of 20 progressed patients or at 24-month post-ADT in 15 of 35 non-progressed patients. The study design and workflow are shown in Figure 1.

### 5hmC sequencing in cfDNA shows high enrichment efficiency and specificity

To determine 5hmC enrichment efficiency and specificity, we spiked in a pool of 180 bp amplicons containing either C, 5mC or 5hmC into cfDNA during library preparation. We first performed PCR analysis in 5hmC-enriched libraries and observed amplicons in 5hmC-containing DNA only (Supplementary Figure S1A). This result was confirmed in the final sequencing libraries, which showed over 100-fold enrichment in reads mapping to 5hmC spike-in DNA (Supplementary Figure S1B–C). We also examined duplication rate in these 5hmC libraries. With a median read count of 18.3 (6.03–42.43) million/sample, the sequencing libraries showed 98% (95–99%) mappable reads and 75% (72–78%) unique (nonduplicate) reads (Supplementary Table S2).

### 5hmC profiles in cfDNA are different from PBMC-derived gDNA

**Figure 2.**
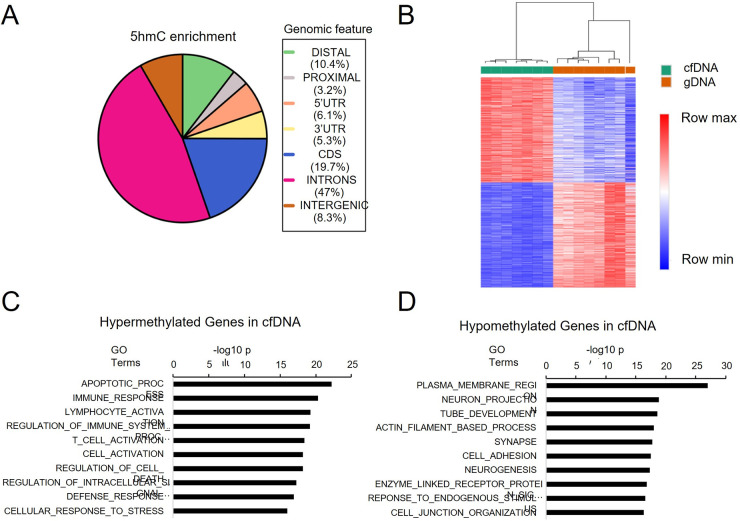
Distribution of 5hmC-enriched regions and genes. A. Genome distribution of 5hmC-enriched regions. B. Heatmap showing differentially methylation genes between cfDNA and blood mononuclear cells-derived gDNA. C. Hallmark gene set enrichment in cfDNA hypermethylated genes. D. Hallmark gene set enrichment in cfDNA hypomethylated genes.

To evaluate the distribution of 5hmC in cfDNA, we first performed enrichment analysis using 1kb window bins across the genome. Peak detection in healthy control cfDNAs showed that 66.7% of 5hmC-enriched regions were located in gene bodies including introns (47%) and CDS (19.7%), whereas only 6.1% enriched regions were found in 5’ UTRs (Figure 2A). We then compared gene body read counts between cfDNA from healthy controls and peripheral blood gDNA. From a total of 23,433 genes with median raw read count ≥8, we observed 11,688 differentially methylated genes (DMGs) including 5,819 hypermethylated genes and 5,869 hypomethylated genes in cfDNA (FDR < 0.1, Figure 2B). Enrichment analysis showed that the hypermethylated DMGs were enriched in apoptotic process and immune response (Figure 2C), whereas hypomethylated genes were enriched in cell structure, cell adhesion and neuron protection (Figure 2D).

### Baseline plasma cfDNAs show diverse 5hmC epigenomic profiles

**Figure 3.**
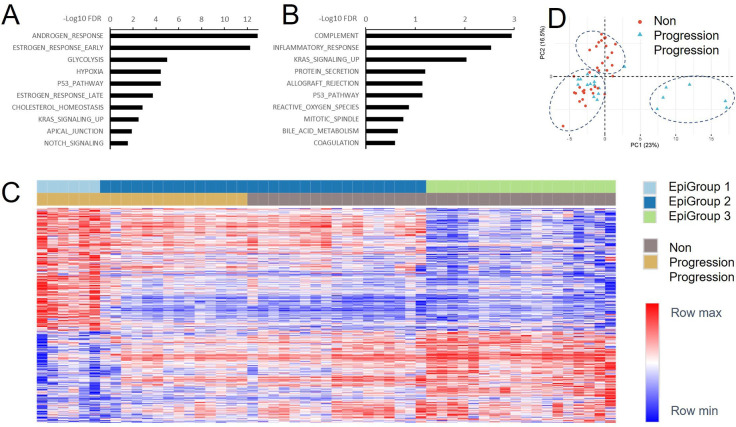
Gene set enrichment and epigenomic heterogeneity. A. Gene set enrichment in hypermethylated genes of baseline samples when comparing progressed to non-progressed patients. B. Gene set enrichment in hypomethylated genes of baseline samples when comparing progressed to non-progressed patients. C. Heatmap showing distinct epigenomic groups in both progressed and non-progressed patients. D. Principal component analysis showing three epigenomic clusters.

To test 5hmC differences at baseline cfDNAs, we compared read count in each individual gene between patients with disease progression (N=20) and those without progression (N=35) during 24-month follow up. Among the 23,433 genes tested, we identified 1,642 DMGs (FDR < 0.1) including 1,008 hypermethylated and 634 hypomethylated genes in the patients with progression (Supplementary Table S3). To evaluate molecular mechanisms underlining the differential methylation, we performed enrichment analysis using these DMGs in hallmark gene sets. This analysis showed significant enrichment in multiple gene sets with androgen response (FDR=1.19E-13) and estrogen response early (FDR=5.50E-13) as top 2 gene sets in hypermethylated genes, and complement (FDR=1.13E-3) and inflammatory response (FDR=2.93E-03) as top 2 gene sets in hypomethylated genes (Figure 3A–B). Interestingly, these DMGs revealed significant epigenomic heterogeneity within both clinical groups of patients. Hierarchical clustering analysis showed that a fraction of progressed patients demonstrated unique epigenomic features that separated them from all other patients although most progressed patients tended to cluster with non-progressed patients (Figure 3C). The same trend was also shown in principal component analysis (Figure 3D). Specifically, although the 55 patients were clinically classified into two groups (progressed and non-progressed), they were epigenetically classified into three groups by unsupervised analysis. EpiGroup 1 included these patients with disease progression (N=6) and showed unique epigenomic features. EpiGroup 2 featured highly similar epigenomic profiles between the subgroups of progressed (N=14) and non-progressed patients (N=17). EpiGroup 3 was a unique cluster of patients without progression (N=18).

### Patients with different epigenomic features show 5hmC hypermethylation in unique signaling pathways

To evaluate molecular mechanisms of the epigenomic heterogeneity, we compared the 5hmC profiles using the 23,433 genes among three EpiGroups. This comparison identified 13,220 (EpiGroups 1 vs. 2), 5,046 (EpiGroups 1 vs. 3) and 12,603 (EpiGroups 2 vs. 3) DMGs (Supplementary Table S4). Gene enrichment analysis showed significant differences in a wide variety of signaling and regulatory pathways. Notably, when compared to either EpiGroup 2 or EpiGroup 3, the EpiGroup 1 consistently showed that the androgen response was the most significantly hypermethylated gene set (FDR≤5.63E-10), while immune responses (complement, allograft rejection and inflammation) were among the top hypomethylated gene sets (FDR≤ 1.04E-4) (Supplementary Figure S2A–D, Table S5). Of note, these hyper- and hypomethylated gene sets had the similar trend to the significant gene sets when comparing two clinical groups (Figure 3A–B). Clearly, EpiGroup 1 is the main driver of epigenomic difference between patients with progression and patients without progression. This unique subgroup is characterized by 5hmC hypermethylation (activation) of the androgen response gene set, and 5hmC demethylation (inactivation) of immune responses. When comparing Epigroups 2 with 3, we observed significant enrichment in hallmark P53 pathway and mitotic spindle gene sets (FDR≤ 2.13E-6) with an increased methylation in EpiGroup 2 (Supplementary Table S5)

### Gene set activity scores in baseline samples classify patients with different clinical outcomes

**Figure 4.**
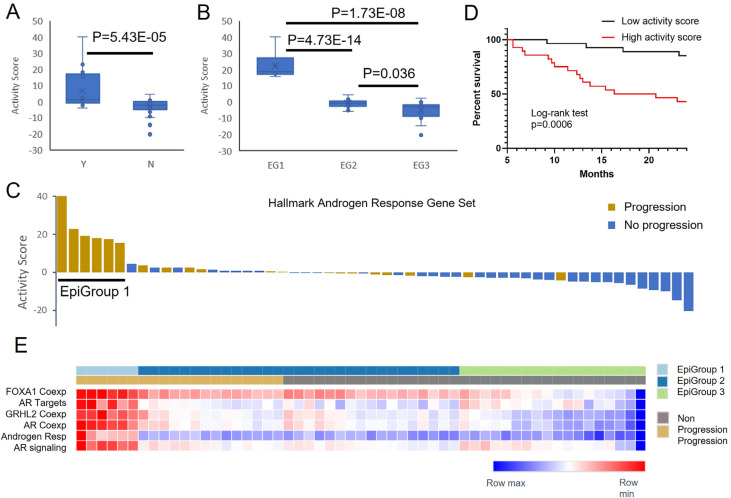
Activity scores in androgen signaling gene sets separate patients with different clinical outcomes. A. Activity score of hallmark androgen response gene set is significantly higher in patients with progression (Y) than without progression (N). B. Activity score in EpiGroup 1 (EG1) is significantly higher than EpiGroups 2 (EG2) and 3 (EG3). C. Waterfall plot shows clear-cut activity score difference in EpiGroup 1. D. High activity score is associated with poor progression-free survival. E. Heatmap of activity scores in six androgen signaling gene sets.

Significant 5hmC enrichment in diverse gene sets indicated a potential role of these genes in treatment response and disease progression during ADT. To quantify the active status of these gene sets, we developed a gene set activity score using a mean value of log2 ratios among all genes of selected gene set. We first calculated the activity score using 97 genes that overlapped with hallmark androgen response gene set. The average activity scores were statistically different (p=5.43E-05) between patients with progression (score= 6.62 ± 11.65) and patients without progression (score= −3.23 ± 4.90) (Figure 4A). Subgroup analysis showed that the difference was driven by EpiGroup 1. Specifically, the average activity scores were 22.27, −0.84 and −4.90 in EpiGroups 1, 2 and 3, respectively. The activity score in EpiGroup 1 was significantly higher than EpiGroup 2 (p=4.73E-14) and EpiGroup 3 (p=1.73E-08) (Figure 4B). Clearly, the patients in EpiGroup 1 had the highest activity scores that were distinguished from the remaining patients regardless of progression status (Figure 4C). Meanwhile, to evaluate whether the activity score was predictive of disease outcome, we performed Kaplan-Meier analysis and observed significant association of the activity score with progression-free survival (p=0.0006, HR= 5.35, Figure 4D). Specifically, comparing to patients with high activity score (median value as cutoff), the patients with low-activity score showed approximately 43% progression-free survival benefit after 24 months of ADT-related treatment.

In addition to hallmark androgen response gene set, we also tested other androgen-related gene sets including *AR* target genes (ChIP-seq), *GRHL2*-coexpressed genes, *FOXA1*-coexpressed genes, *AR*-coexpressed genes [39] and *AR* signaling pathway [40]. Overall distribution of these activity scores in the selected gene sets are shown in Figure 4E. To test the activity differences, we first compared two groups of patients with different clinical outcomes in baseline samples. This comparison showed significantly higher activity scores in patients with progression than those without progression (Supplementary Figure S3A). For example, the activity score in *AR*-coexpressed gene set was significantly higher in progressed than non-progressed patients (p=1.45E-05). We then compared three groups of patients with different epigenomic features. This analysis showed consistently higher activity scores in EpiGroup 1 than EpiGroups 2 and 3 patients. In 4 of 6 androgen-related gene sets (hallmark androgen response, *GRHL2*-, *FOXA1*- and *AR*-coexpressed gene sets), EpiGroup 2 also showed higher activity score than EpiGroup 3 (Figure 4B, Supplementary Figure S3B). Kaplan-Meier analysis also demonstrated poor progression-free survival in patients with high activity score (Supplementary Figure S3C). Clearly, multiple gene sets involved in androgen responses are hypermethylated (activated) before initiating ADT in the patients with disease progression, in particular, in the patients of EpiGroup 1.

### Baseline gene set activity score predicts treatment resistance independent of clinical factors and androgen receptor amplification

Since multiple clinical factors have shown a potential association with disease progression, we tested whether these clinical factors had any effect on discriminative performance of the gene set activity scores. We first tested Gleason score at diagnosis, age at enrollment, metastatic volume, and baseline PSA for their potential association with disease progression in the 55 patients. Among all clinical factors tested, only PSA level at enrollment showed significant association with disease progression (p=2.35E-05) (Supplementary Figure S4A). Because the whole genome 5hmC sequencing data can be used for estimating ctDNA fraction [29], we then calculated the ctDNA percentage using a previously published algorithm [41]. We found that the higher baseline ctDNA percentage was also associated with disease progression (p=0.0035) (Supplementary Figure S4B). Because of their clinical associations, we further performed analysis of covariance to re-evaluate the differences of activity scores in the patients with different clinical outcomes. By applying baseline PSA level and ctDNA percentage as covariates, our analysis showed that baseline activity scores in patients with disease progression were still significantly higher than the ones without progression in 4 of six gene sets. More importantly, the baseline activity scores in EpiGroup 1 (part of progressed patients) remained significantly higher than other patients regardless of progression status (Supplementary Table S6).

Additionally, we also evaluated 5hmC status at *AR* locus. We plotted the mean values of read count log2 ratios in the genes flanking *AR* and observed significantly higher log2 ratios in EpiGroup 1 than EpiGroups 2 and 3 at *AR* locus. However, flanking gene loci did not show significantly high read count in the EpiGroup 1 (Supplementary Figure S4C). Since the 5hmC capture assay is highly specific, the high level of 5hmC at *AR* but not nearby gene loci indicate specific activation of *AR* expression. Additionally, the *AR* is commonly co-amplified with nearby genes [42, 43], the lack of co-amplification also supports the notion that the activation of androgen response gene sets may not be caused by *AR* amplification in this study cohort.

### Dynamics of gene set activity scores reflects treatment response and serves as surrogates to monitor disease progression

To evaluate whether 5hmC profile changes reflect treatment outcomes, we compared dynamics of the entire 23,433 genes at different blood collection times in three groups of patients with different epigenomic features. The blood collection times include baseline, 3-month, and either at progression or at 24-month if no progression. Although patients in EpiGroup 2 did not show significant difference between different timepoints, we did observe significant changes when comparing 3-month to baselines in EpiGroup 1 and EpiGroup 3. Specifically, 3-month treatment caused significant 5hmC changes in 4,315 genes including 2,627 reduced and 1,688 increased methylations in EpiGroup 1, and 4,112 genes including 1,803 reduced and 2,309 increased methylation in EpiGroup 3 (FDR<0.1, Supplementary Table S7). Interestingly, enrichment analysis in EpiGroup 1 showed the most significant methylation reduction in gene sets of androgen and estrogen responses while the most significant methylation increases in the gene sets of immune responses (Supplementary Table S8). Since the EpiGroup 1 inherently showed high level of 5hmC methylation in androgen-related pathways at baseline, reduction of 5hmC in these gene sets at 3-month treatment may reflect effective suppression of androgen pathway activity by ADT. Similarly, enrichment analysis in EpiGroup 3 showed that 3-month treatment induced significant methylation increases in gene sets of mitotic spindle and p53 pathway (Supplementary Table S9) although we did not observe significant methylation reduction in any hallmark gene sets.

**Figure 5.**
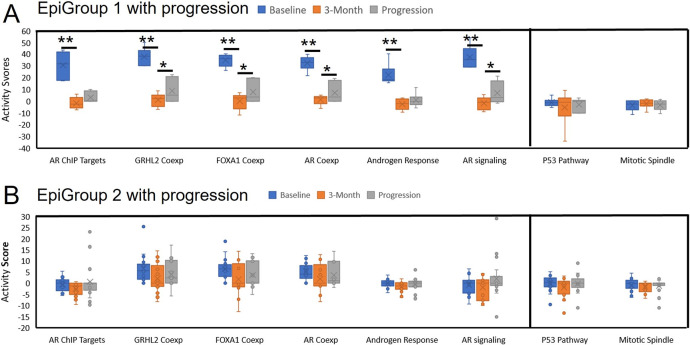
Dynamic changes of activity scores in patients with disease progression during ADT. A. Activity scores in EpiGroup 1 patients were significantly reduced from baseline to 3-month but returned to higher level when disease progressed in all six androgen signaling gene sets, but not in P53 and mitotic spindle pathways. B. Activity scores in EpiGroup 2 patients did not show significant changes in all gene sets tested. Two stars (**) indicate p value<0.0001 when compared 3-month to baseline. One star (*) represents p value < 0.05 when compared at progression to 3-month.

We also compared dynamics of gene set activity scores at three blood collection times. When compared to baseline, 3-month treatment in EpiGroup 1 patients significantly reduced activity scores in all androgen response gene sets (p<0.0001). Importantly, these reduced activity scores were then significantly increased to a higher level in 4 of the 6 androgen-related gene sets when disease progressed (p<0.05) (Figure 5A). However, these dynamic changes were not evident in EpiGroup 2 and EpiGroup 3 although some differences were significant (Figure 5B, Supplementary Figure S5A–B). Since 3-month treatment in EpiGroup 3 also induced significant increase of 5hmC in gene sets of mitotic spindle and p53 pathway, we also analyzed dynamics of their activity scores in these gene sets. As expected, the activity scores in both gene sets were increased in the EpiGroup 3 when comparing 3-month to baseline (p<0.02, Supplementary Figure S5A). Meanwhile, we also observed a decrease of these activity score in EpiGroup 2 patients without disease progression (Supplementary Figure S5B). This reduced activity score in EpiGroup 2 was also expected due to relatively higher 5hmC level of the two gene sets in EpiGroups 2 than 3 (Supplementary Table S5). At 24-month, the activity scores of mitotic spindle and p53 pathway remained at the level similar to 3-month. In EpiGroup 1 patients and EpiGroup 2 patients with disease progression, however, we did not observe any changes in mitotic spindle and p53 pathway among the three timepoints (Figure 5A–B).

## Discussion

Recent studies have demonstrated that 5hmC, a relatively stable intermediate product of active DNA demethylation, plays a critical role in gene expression regulation and is also regarded as a novel epigenomic biomarker for cancer diagnosis and prognosis[29, 30, 44, 45]. In this study, we sought to evaluate clinical utility of 5hmC in cfDNA as minimally invasive biomarkers for outcome prediction in hormone-sensitive prostate cancer patients undergoing ADT. This study shows that 5hmC gains in specific gene sets are significantly associated with adverse outcome in these patients. Activity scores from these gene sets can separate a subgroup of patients who develop early resistance to the treatment from those benefiting from a longer treatment response time. Our study further demonstrated three distinct epigenomic subgroups in this patient cohort with one subgroup featuring preexisting activation of androgen response gene sets and early treatment failure. Serial time point analysis shows that dynamics of 5hmC profiles reflect treatment response and may serve as sensitive biomarker to monitor disease progression during the ADT.

Currently, combining ADT with LHRH analog and ARSI with or without docetaxel is the standard of care for newly diagnosed metastatic castrate-sensitive prostate cancer [46]. Although there are no head-to-head comparisons, multiple published meta-analysis did not show statistically significant improvement in overall survival with adding docetaxel to LHRH analog and ARSI (triple therapy) compared to LHRH analog plus ARSI (doublet therapy). Post hoc analysis of the ARASENS trial indicate the overall survival benefit are mainly in high volume but not for low volume metastatic castrate-sensitive prostate cancer[47, 48]. Retrospective biomarker studies have reported prognostic biomarkers like mutations in *TP53* or *RB*; and the potential predicative value of luminal versus basal subtype to combined ADT. However, these biomarkers have not been validated in prospective studies. Our 5hmC study demonstrated that prostate cancer patients receiving ADT can be classified into at least 3 epigenomic subgroups. The subgroup with pre-existing androgen signaling activation had shorter time to become castration resistant. Further validation of this finding in a larger patient population will facilitate discovery of more effective biomarker in future clinical trial.

One novel finding from this study was the identification of significant 5hmC enrichment of androgen response gene sets in patients with earlier disease progression on ADT. Importantly, this enrichment was present in baseline samples of subgroup patients, suggesting potential use for identifying patients who would benefit from upfront treatment intensification. In fact, all patients in this subgroup (N=6) received doublet therapy (4 cases for ADT + docetaxel and 2 cases for ADT + ARSI). Although initial treatment seems effective (for example, the reduced PSA), this group of patients quickly showed disease progression within one year (median PFS=11.73 months). This result suggests inherent resistance to the doublet therapy in this subgroup of patients who featured pre-activation of androgen response genes. Furthermore, activation signal was not limited to hallmark androgen response gene set. We also observed the activation signals from the coexpressed gene sets of *AR*, *FOXA1* and *GRHL2*. Meanwhile, the gene set involved in *AR*-binding targets also showed activation in the subgroup of patients. This observation is consistent with a recent study showing that 5hmC marks the activation of major driver genes in advanced prostate cancer, not only the gene bodies but also downstream target binding sites [25]. Clearly, these activity scores not only reflect driver gene activation but also are predictive of disease progression. With further validation, the activity scores may have potential as biomarkers to stratify patients in future androgen-targeted clinical trials.

The 5hmC epigenomic changes are highly tissue specific and are more frequently found in genes that drive tissue differentiation and among tissue-specific transcription factors [49, 50]. It is more reasonable to speculate the high level of 5hmC in androgen signaling gene sets from prostate cancer origin. However, the current study does not have direct evidence to support that the epigenomic signals were derived from prostate cancer cells only. For example, our study shows significant enrichment of both *FOXA1*- and *GRHL2*-coexpressed genes in patients with disease progression. Using whole blood as RNA source, however, a study found that higher *GRHL2* RNA expression was associated with poor survival in metastatic hormone-sensitive and castration-resistant prostate cancer [51], suggesting that the 5hmC enrichment of *GRHL2* in cfDNA may be derived from blood cells in high risk patients. In contrast, the same study did not observe any association between *FOXA1* RNA expression in blood and poor prognosis. Therefore, the high 5hmC levels in these gene sets are more likely to originate from combination of tumor and nontumor cells. Nevertheless, the gene set activity scores seem effectively separate patients with disease progression from those without.

In addition to predictive value, we also found that the 5hmC dynamics in these gene sets may be used to monitor treatment response and disease progression. Our study shows that higher activity scores are significantly reduced in a subgroup of patients with activation in androgen signaling genes. However, this score reduction does not last long before returning to higher levels at disease progression. In addition to androgen signaling, we also observe an increased activity scores of P53 and mitotic spindle pathways in another subgroup patients during the ADT, suggesting activation of the p53-mediated stress response, and cell cycle regulation. These epigenomic changes may reflect the therapeutic effects and influence treatment response and disease progression. Meanwhile, we also observed significant 5hmC changes after receiving ADT in gene sets related to immune response pathways in a subgroup of patients. Clearly, the changes of the activity scores during treatment are coincident with treatment response and progression. To our knowledge, this is the first study to demonstrate potential applications of 5hmC-based cfDNA enrichment analysis in predicting treatment response and monitoring disease progression in patients with hormone sensitive prostate cancer.

Although this study provided strong evidence showing association of the epigenomic modifications with resistance to ADT, the current study also has some limitations. First, the study cohort was collected from 2015 to 2019 when the standard care was in transition from ADT to doublet or triplet therapy. Therefore, the study cohort has a significant number of patients (23 of 55) receiving ADT alone. Second, the study cohort is relatively small, which does not allow more detail analysis by further stratifying patients based on their treatment strategies and ethnical groups. Third, this initial discovery was from one cohort only. Lack of an independent validation cohort may lead to cohort-dependent biases. Last, the activation of androgen response gene sets was found in a fraction of patients with early resistance to ADT. Other factors affecting early treatment failure remain unknown. Nevertheless, our study is an initial step to develop an epigenome-based liquid biopsy tool for more effective treatment of the hormone-sensitive prostate cancer.

## Conclusions

Our genome-wide 5hmC sequencing in cfDNA revealed unique epigenomic signatures in hormone-sensitive prostate cancer patients receiving ADT. These unique 5hmC profiles can identify the patients likely to develop earlier resistance to ADT. Notably, we identified a subgroup of patients featuring pre-activation of androgen response gene sets before initiating ADT. Since the gene set activation is a main driver to confer treatment resistance, we proposed a 5hmC-based gene set activity score algorithm to quantify the activation status and serve as a biomarker for prediction of treatment response and monitor disease progression.

## Supplementary Material

Supplement 1

Supplement 2

## Figures and Tables

**Table 1.** Clinical characteristics of patients

Total Number		55

**Age**		
	Median	69
	Range	49-94
**Race**		
	Caucasian	44
	African American	9
	Others	2
**Gleason score at initial diagnosis**		
	6	2
	7	21
	>=8	30
	Missing	2
**Baseline Prostate-Specific Antigen (ng/ml)**		
	Median	21.5
	Range	0.29-3275
**Metastases to bone or soft tissues**		
	Yes	44
	No	11
**Metastatic volume**		
	High	18
	Low	26
	No	11
**Radical prostatectomy (RP) and radiotherapy (RT)**		
	RP only	7
	RT only	8
	RP+RT	12
	None of RP and RT	28
**Progression within 24-month follow up**		
	Yes	20
	No	35
**Time from ADT start to ADT failure (months)**		
	Median	12.53
	Range	5.6-23.2
