# Supplementary material for: 5-hydroxymethylcytosine sequencing in plasma cell-free DNA identifies unique epigenomic features in prostate cancer patients resistant to androgen deprivation therapy
Source: medRxiv. 2023 Oct 16:2023.10.13.23296758. Preprint. [Version 1] doi: 10.1101/2023.10.13.23296758 (PMC10615016; doi:10.1101/2023.10.13.23296758)
Supplement: Supplement 2 [file NIHPP2023.10.13.23296758v1-supplement-2.pdf]

medRxiv preprint doi: https://doi.org/10.1101/2023.10.13.23296758; this version posted October 16, 2023. The copyright holder for this preprint (which was not certified by peer review) is the author/funder, who has granted medRxiv a license to display the preprint in perpetuity.
It is made available under a CC-BY 4.0 International license .

**Supplementary Figure S1. Enrichment efficiency and specificity.** A. PCRs using spike-in templates from non-enriched and enriched DNAs showed specific capture of 5hmC-containing fragments. B. Read count percentages of spike-in DNA in a non-enriched sequencing library. C. Read count percentages of spike-in DNA in an enriched sequencing library.

**Supplementary Figure S2. Gene set enrichment analysis in differentially methylated genes.** EpiGroup 1 showed significant hypermethylation in the androgen response gene set and significant hypomethylation in immune responses (complement, allograft rejection and inflammation) gene sets when compared to EpiGroup 2 (A, B) and Epigroup 3 (C, D).

**Supplementary Figure 3. Activity score differences between patients with different clinical outcomes or among patients with different epigenetic status** Significant differences between patients with different outcomes or among patients with different epigenetic status. Boxplots are shown in A (progressed vs nonp-rogressed patients) and in B (three EpiGroups). Progression-free survival analyses are shown in C.

**Supplementary Figure 4. Effect of PSA and ctDNA percentage on clinical outcome and distribution of gene activity at AR locus.** A. Baseline PSA is significantly higher in progressed patients (Y) than non-progressed patients (N). B. ctDNA percentage is significantly higher in progressed patients (Y) than non-progressed patients (N). C. Distribution of read count log2 ratios shows 5hmC enrichment at AR locus but not at AR flanking loci.

**Supplementary Figure 5. Dynamic changes of activity scores in patients without disease progression during ADT.** A. Activity scores in EpiGroup 3 patients show significant increases in 3 of six androgen signaling gene sets as well as P53 and mitotic spindle gene sets. B. Activity scores in EpiGroup 2 patients were significantly reduced from baseline to 3-month in 2 of six androgen signaling gene sets and mitotic spindle gene set. The star (*) indicates p value <0.05 when compared 3-month to baseline.

**Supplementary Table S1.** Detail clinical characteristics of patients

**Supplementary Table S2.** Total reads, mappable reads and unique reads

medRxiv preprint doi: https://doi.org/10.1101/2023.10.13.23296758; this version posted October 16, 2023. The copyright holder for this preprint (which was not certified by peer review) is the author/funder, who has granted medRxiv a license to display the preprint in perpetuity.
It is made available under a CC-BY 4.0 International license .

**Supplementary Table S3.** Differentially methylated genes (DMGs) between disease progression and non-progression patients

**Supplementary Table S4.** Differentially methylated genes (DMGs) among three EpiGroups

**Supplementary Table S5.** Gene set enrichment analysis among different EpiGroups

**Supplementary Table S6.** Analysis of Covariance in patients with different clinical outcomes and different EpiGroups

**Supplementary Table S7.** Dynamics of activity scores in different EpiGroups

**Supplementary Table S8.** Gene set enrichment analysis in EpiGroup 1 showing significant methylation decreases in estrogen and androgen response genes and increase in immune responses after 3-month treatment

**Supplementary Table S9.** Gene set enrichment analysis in EpiGroup 3 showing significant methylation increases in gene sets of mitotic spindle and p53 pathway after 3-month treatment

medRxiv preprint doi: https://doi.org/10.1101/2023.10.13.23296758; this version posted October 16, 2023. The copyright holder for this preprint (which was not certified by peer review) is the author/funder, who has granted medRxiv a license to display the preprint in perpetuity.
It is made available under a CC-BY 4.0 International license .

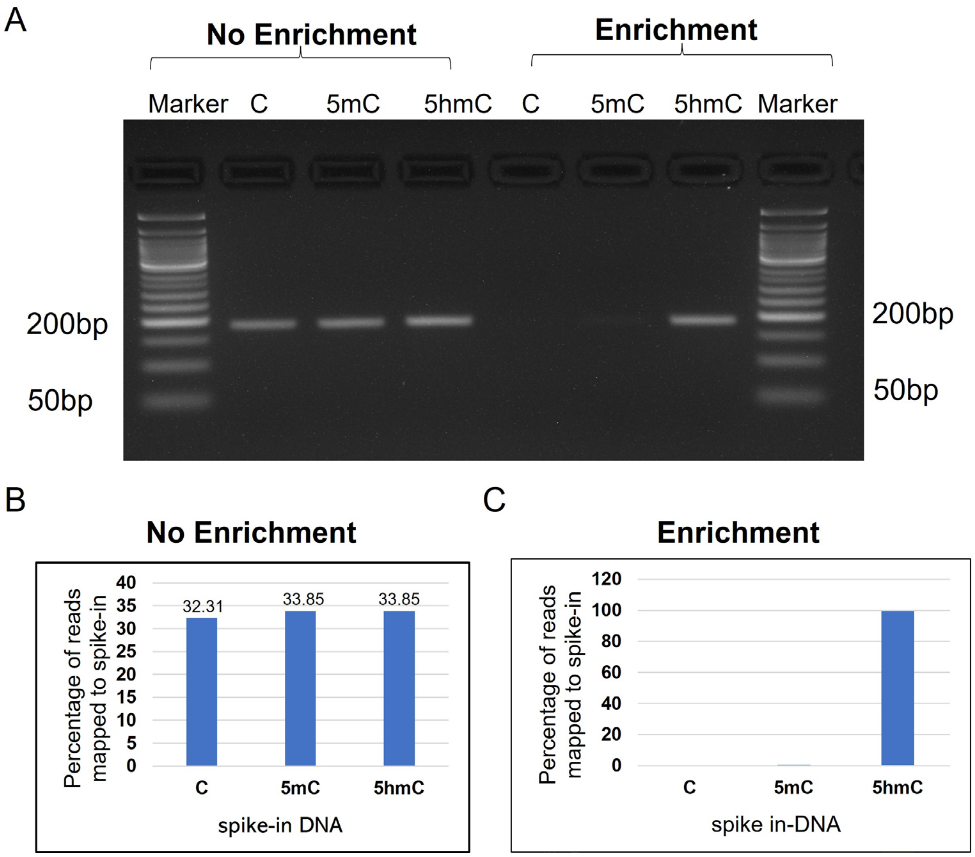


**Supplementary Figure S1. Enrichment efficiency and specificity.** A. PCRs using spike-in templates from non-enriched and enriched DNAs showed specific capture of 5hmC-containing fragments. B. Read count percentages of spike-in DNA in a non-enriched sequencing library. C. Read count percentages of spike-in DNA in an enriched sequencing library.

medRxiv preprint doi: https://doi.org/10.1101/2023.10.13.23296758; this version posted October 16, 2023. The copyright holder for this preprint (which was not certified by peer review) is the author/funder, who has granted medRxiv a license to display the preprint in perpetuity.
It is made available under a CC-BY 4.0 International license .

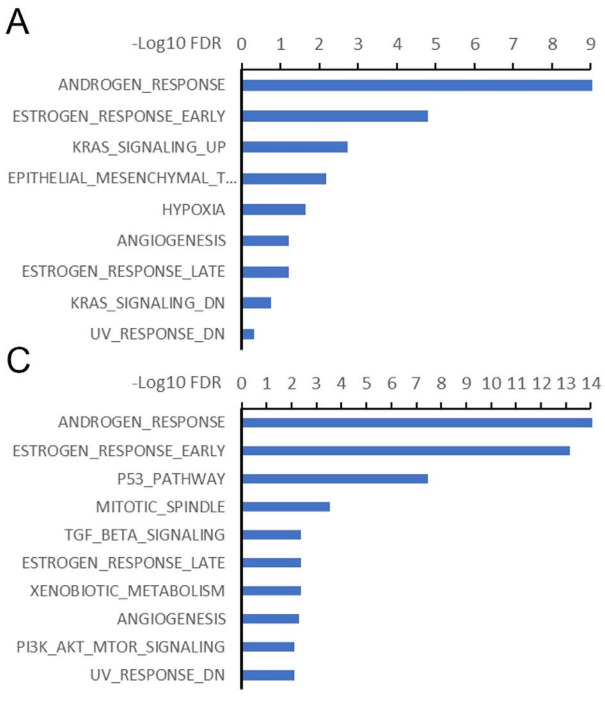


**Supplementary Figure S2. Gene set enrichment analysis in differentially methylated genes.** EpiGroup 1 showed significant hypermethylation in the androgen response gene set and significant hypomethylation in immune responses (complement, allograft rejection and inflammation) gene sets when compared to EpiGroup 2 (A, B) and Epigroup 3 (C, D).

medRxiv preprint doi: https://doi.org/10.1101/2023.10.13.23296758; this version posted October 16, 2023. The copyright holder for this preprint (which was not certified by peer review) is the author/funder, who has granted medRxiv a license to display the preprint in perpetuity.
It is made available under a CC-BY 4.0 International license .

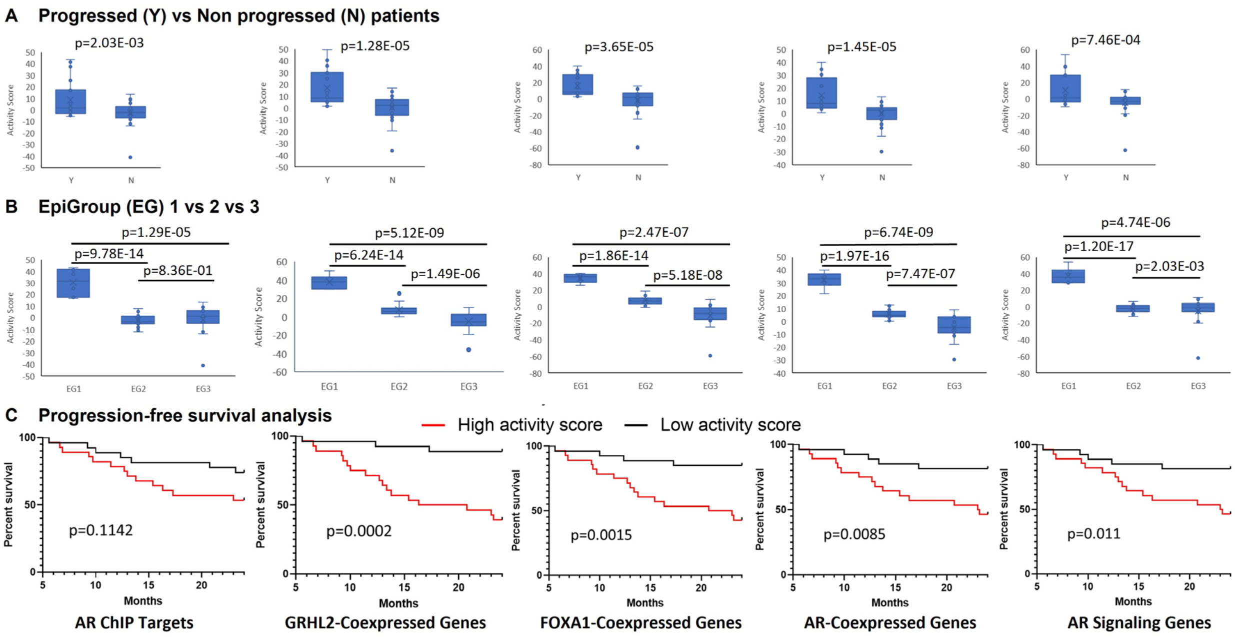


**Supplementary Figure 3. Activity score differences between patients with different clinical outcomes or among patients with different epigenetic status**

Significant differences between patients with different outcomes or among patients with different epigenetic status. Boxplots are shown in A (progressed vs nonprogressed patients) and in B (three EpiGroups). Progression-free survival analyses are shown in C.

medRxiv preprint doi: https://doi.org/10.1101/2023.10.13.23296758; this version posted October 16, 2023. The copyright holder for this preprint (which was not certified by peer review) is the author/funder, who has granted medRxiv a license to display the preprint in perpetuity.
It is made available under a CC-BY 4.0 International license .

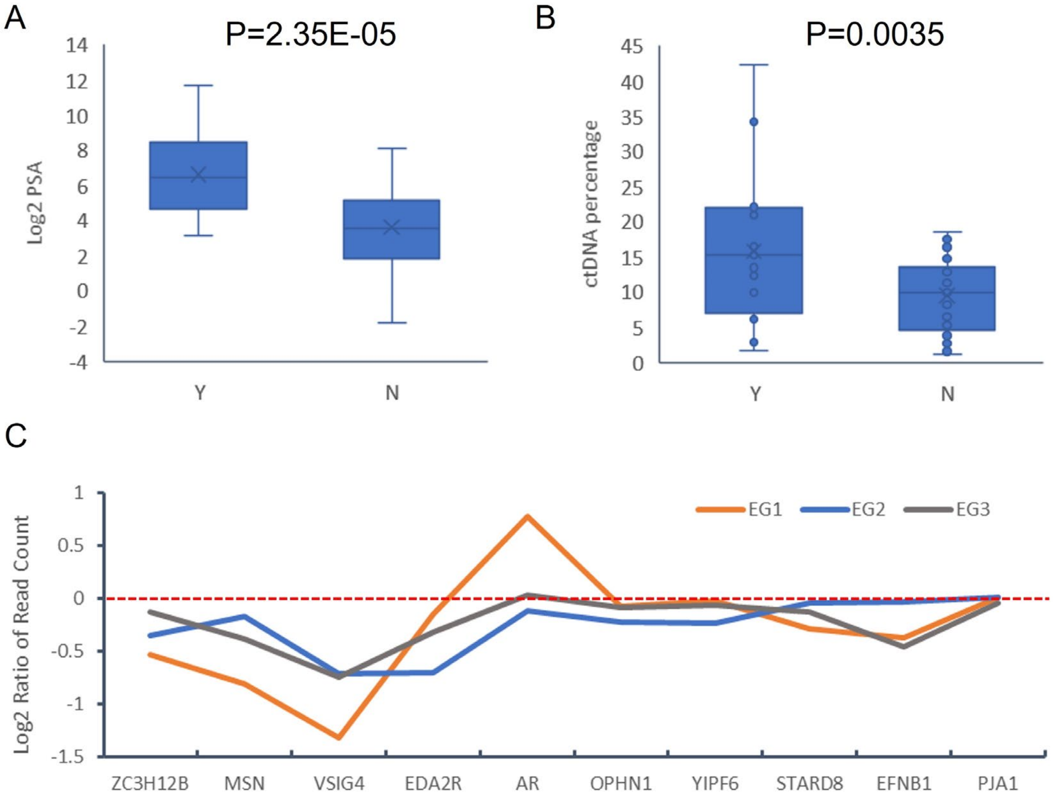


**Supplementary Figure 4. Effect of PSA and ctDNA percentage on clinical outcome and distribution of gene activity at AR locus.** A. Baseline PSA is significantly higher in progressed patients (Y) than non-progressed patients (N). B. ctDNA percentage is significantly higher in progressed patients (Y) than non-progressed patients (N). C. Distribution of read count log2 ratios shows 5hmC enrichment at AR locus but not at AR flanking loci.

medRxiv preprint doi: https://doi.org/10.1101/2023.10.13.23296758; this version posted October 16, 2023. The copyright holder for this preprint
(which was not certified by peer review) is the author/funder, who has granted medRxiv a license to display the preprint in perpetuity.
It is made available under a CC-BY 4.0 International license .

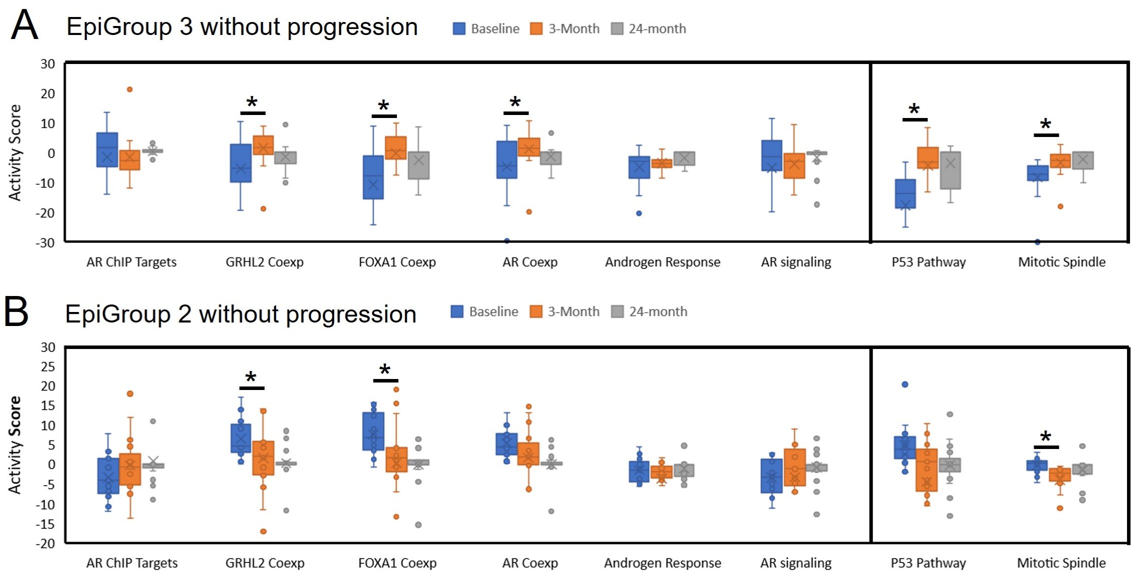


**Supplementary Figure 5. Dynamic changes of activity scores in patients without disease progression during ADT.** A. Activity scores in EpiGroup 3 patients show significant increases in 3 of six androgen signaling gene sets as well as P53 and mitotic spindle gene sets. B. Activity scores in EpiGroup 2 patients were significantly reduced from baseline to 3-month in 2 of six androgen signaling gene sets and mitotic spindle gene set. The star (*) indicates p value <0.05 when compared 3-month to baseline.
